# Clinical characteristics and prognosis of patients with hypertrophic cardiomyopathy and heart failure with preserved ejection fraction

**DOI:** 10.1007/s00392-023-02371-5

**Published:** 2024-01-10

**Authors:** Qin-Fen Chen, Jiandong Hu, Jie Hu, Prabhjot S. Nijjar, Jiahui Xu, Shanzhen Shi, Dongjie Liang, Hetong Liao, Jiaqi Gao, Wei-Hong Lin, Shenban You, Xiao-Dong Zhou

**Affiliations:** 1 Medical Care Center, The First Affiliated Hospital of Wenzhou Medical University, Nanbaixiang, Wenzhou, 325100 China; 2 Institute of Aging, Key Laboratory of Alzheimer’s Disease of Zhejiang Province, Wenzhou Medical University, Wenzhou, 325000 China; 3 Department of Information, First Affiliated Hospital of Wenzhou Medical University, Wenzhou, 325000 China; 4 Department of Cardiovascular Medicine, The Heart Center, The First Affiliated Hospital of Wenzhou Medical University, Wenzhou, 325000 China; 5 Division of Cardiovascular Medicine, Department of Medicine, University of Minnesota Medical School, Minneapolis, MN USA

**Keywords:** Heart failure with preserved ejection fraction, Heart failure with reduced ejection fraction, Hypertrophic cardiomyopathy, End-stage heart failure, Prognosis

## Abstract

**Background:**

Whether heart failure with preserved ejection fraction (HFpEF) is associated with an increased risk of developing systolic dysfunction and a poor prognosis in hypertrophic cardiomyopathy (HCM) patients is unknown.

**Objective:**

We aimed to assess risk factors for the development of end-stage (ES) heart failure (HF) (ejection fraction < 50%) and compare the prognosis of different HF phenotypes.

**Methods:**

This retrospective study was conducted on patients with HCM in China between January 2009 and February 2023. Patients were stratified into three different groups: HCM-non-HF, HCM-HFpEF and HCM-heart failure with reduced ejection fraction (HCM-HFrEF). The primary outcome was a composite of major adverse cardiac events (MACEs), including all-cause deaths, HF hospitalization, sudden cardiac death and ventricular tachycardia.

**Results:**

Of 3,620 HCM patients enrolled, 1,553 (42.9%) had non-HF, 1,666 (46.0%) had HFpEF, and 579 patients (11.1%) had HFrEF at baseline. During the median follow-up period of 4.0 years (IQR 1.4–9.4 years), patients with HCM-HFpEF exhibited a higher incidence of ES-HF than those with HCM-non-HF (12.4% vs. 2.7%, *P* < 0.001). HFpEF was an independent risk factor for ES-HF development (HR 3.84, 2.54–5.80, *P* < 0.001). MACEs occurred in 26.9% with a higher incidence in HCM-HFpEF than HCM-non-HF (36.6% vs 12.2%, *P* < 0.001). HFpEF was an independent predictor of MACEs (HR 2.13, 1.75–2.59, *P* < 0.001).

**Conclusions:**

HFpEF is common in HCM. Compared to non-HF, it increases the risk of LVEF decline and poor prognosis. It may aid in risk stratification and need close echocardiography follow-up.

**Graphical Abstract:**

Clinical Characteristics and Prognosis of Patients with Hypertrophic Cardiomyopathy and Heart Failure with Preserved Ejection Fraction. *Abbreviations:* ES-HF: end-stage heart failure; HCM: hypertrophic cardiomyopathy; HFpEF: heart failure with preserved ejection fraction; MACEs: major adverse clinical events

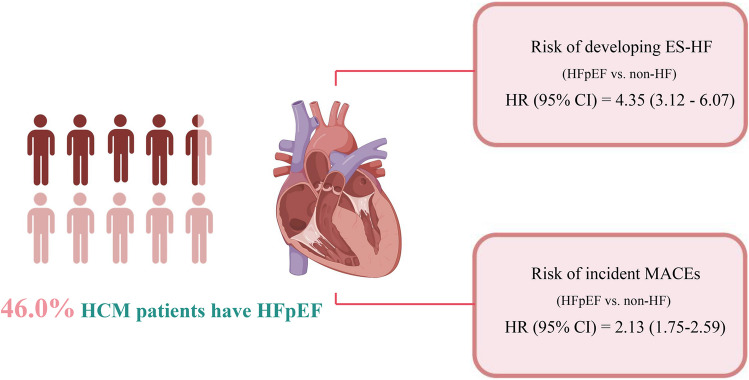

**Supplementary Information:**

The online version contains supplementary material available at 10.1007/s00392-023-02371-5.

## Introduction

Hypertrophic cardiomyopathy (HCM) is a genetic cardiac disorder characterized by abnormal thickening of the myocardium, with an estimated prevalence of 1 in 500 individuals worldwide [1–3]. It is a common cause of sudden cardiac death (SCD) and heart failure (HF) in young individuals [4–6]. HCM can present with a wide range of symptoms, but one of the most common complications is heart failure with preserved ejection fraction (HFpEF), also known as diastolic HF [7–9].

HFpEF is a clinical syndrome characterized by symptoms and signs of HF in the absence of significant left ventricular systolic dysfunction [10–12]. HFpEF is associated with high morbidity and mortality rates, though limited research has focused on the prognosis of patients with HCM and HFpEF [13, 14]. A subset of patients with HCM may develop end-stage HF (ES-HF), which refers to the advanced stage of HF characterized by systolic dysfunction, defined as left ventricular ejection fraction (LVEF) < 50% [6, 15]. ES-HF often requires advanced HF therapies and is associated with poor prognosis and quality of life [16–18]. Therefore, it is imperative to identify risk factors for the development of ES-HF. Whether HFpEF increases the risk of progression to ES-HF, and the impact of these different HF phenotypes on prognosis in HCM remains unclear.

Understanding the differences in prognosis among these patient groups is crucial for tailoring appropriate management strategies and improving patient outcomes in HCM [19]. Thus, in a large cohort of patients with HCM we aimed to assess the incidence of ES-HF in patients with HCM-HFpEF and HCM-non-HF, and compared the prognosis of patients with HCM-non-HF, HCM-HFpEF and HCM-heart failure with reduced ejection fraction (HCM-HFrEF).

## Methods

### Study design

This retrospective study included patients who were diagnosed with HCM at the First Affiliated Hospital of Wenzhou Medical University from January 2009 to February 2023. Patients were identified from the inpatient admission records. We conducted a thorough search of electronic medical records for 963,201 consecutive transthoracic echocardiograms (TTEs) reports using the specific keyword "hypertrophic cardiomyopathy." We initially screened all patients who had reported HCM at hospitalization but excluded those with incomplete TTE or poor image, missing baseline echocardiography data, no clinical or echocardiography follow-up data, and follow-up time is < 30 days. We obtained baseline information from the electronic medical records such as demographic features, medical history, medication at discharge, echocardiographic evaluation, and follow-up data. The study was conducted following the Declaration of Helsinki. The research protocol was approved by the ethics committee of the First Affiliated Hospital of Wenzhou Medical University, with a waiver of informed consent. The corresponding author, zhouxiaodong@wmu.edu.cn, can provide access to the data supporting the findings upon reasonable request.

### Study definitions

HCM was diagnosed according to guideline recommendations based on Echocardiography or Cardiac Magnetic Resonance imaging (CMR) (left ventricular hypertrophy with a wall thickness of ≥ 15 mm, or ≥ 13 mm in patients with a family history of HCM) in the absence of any other causes of hypertrophy, such as uncontrolled hypertension, cardiac valve disease, and phenocopies [20, 21]. The diagnosis of HFpEF was defined as symptomatic patients with ‘preserved’ ejection fraction (LVEF ≥ 50%) who had at least one of the following conditions: evidence of structural heart disease (including left atrial enlargement) and/or diastolic dysfunction, multiple cardiovascular risk factors with elevated levels of serum natriuretic peptides, or persistently elevated cardiac troponins, in the absence of competing diagnoses [22, 23]. Patients with HCM-nonHF should not manifest any symptoms or display any of the conditions mentioned above. The assessment of symptoms, such as breathlessness and fatigue, was primarily based on self-assessed symptoms and physical examination conducted by the clinician at the time of admission. Based on expert consensus guidelines in HCM, ES-HF was defined as LVEF < 50% as determined by 2-dimensional Echocardiography [4, 7]. Three subgroups are identified: (1) patients with HCM who have preserved ejection fraction and no clinical symptoms of heart failure (HCM-non-HF), (2) patients with HCM who have preserved ejection fraction and clinical symptoms of heart failure (HCM-HFpEF), and finally (3) patients with HCM who have reduced ejection fraction (LVEF < 50%) and clinical symptoms of heart failure (HCM-HFrEF).

Follow-up data were collected from inpatient and outpatient medical records. The follow-up period was the time between HCM diagnosis and either the occurrence of the final clinical follow-up or the date of death, whichever came first. Patients were followed up until April 2023. The primary outcome was a composite of major adverse cardiac events (MACEs), including all-cause deaths, HF hospitalization, SCD and ventricular tachyarrhythmia (VT). SCD is defined as a sudden pulseless condition resulting from a cardiac cause in a previously stable individual. VT is defined as episodes of ventricular tachycardia or ventricular fibrillation that require implantable cardioverter-defibrillator (ICD) therapies or untreated ventricular tachyarrhythmia episodes that last for more than 30 s and are detected by the ICD.

### Statistical analysis

Normally distributed continuous variables were presented as mean ± standard deviation (SD), while non-normally distributed continuous variables were presented as median with interquartile range (IQR). Categorical variables were presented as the number (%) of patients. The comparison between groups was conducted using Student's t-test (for normally distributed continuous variables), Mann-Whitney U-test (for non-normally distributed continuous variables), and Chi-squared test (for categorical variables). Additionally, a Cox proportional hazards model was developed to evaluate the effect of different HF phenotypes on clinical outcomes. Variables for inclusion in the multivariable analysis were decided a priori based on known confounders. Hazard ratios (HR) and 95% confidence intervals (CI) were calculated. Event-free survival curves were computed using the Kaplan-Meier method, and differences between the curves were compared using the log-rank test. A significance level of *P* < 0.05 (two-tailed) was considered statistically significant. All statistical analyses were carried out using the 23.0 version of IBM SPSS software for Windows.

## Results

### Baseline characteristics

**Fig. 1:**
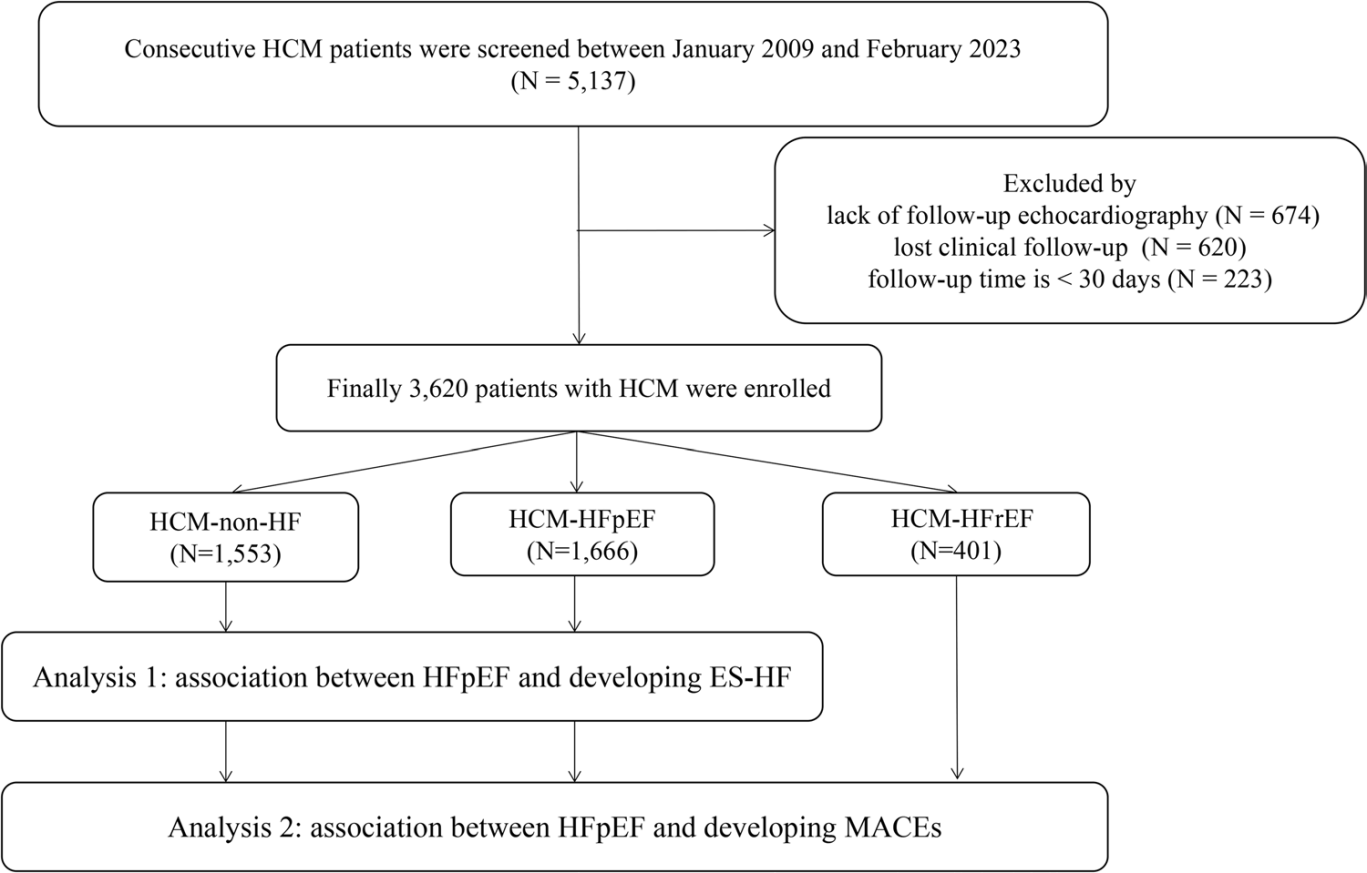
Flow diagram. *Abbreviations:* ES-HF: end-stage heart failure; HF: heart failure; HCM: hypertrophic cardiomyopathy; HFpEF, heart failure with preserved ejection fraction; HFrEF: heart failure with reduced ejection fraction

From 5137 consecutive patients diagnosed with HCM, 1517 were excluded due to lack of follow-up Echocardiogram (*n* = 674), loss to follow-up (*n* = 620) and follow-up period < 30 days (*n* = 223) (Fig. 1). The final study sample consisted of 3,620 patients with HCM. A total of 3620 patients were diagnosed through echocardiography, whereas 326 patients underwent cardiac magnetic resonance (CMR) for diagnosis. The diagnosis of HCM-HFpEF patients was based on only left atrial (LA) enlargement in 229 individuals, LA enlargement coupled with elevated levels of B-type natriuretic peptide (BNP) in 450 individuals, and abnormal E/e' ratio in 1361 individuals. At baseline, 1,553 (42.9%) had HCM-non-HF, 1,666 (46.0%) had HCM-HFpEF, and only 401 (11.1%) had HCM-HFrEF. Detailed baseline characteristics of the study population, stratified by the 3 HF groups, are shown in Table 1. The mean age of the study population was 61.4 ± 14.0 years, and the prevalence of males was 67.4%. Patients with HFrEF were more likely to be male compared to those with HFpEF or non-HF (79.8% compared to 64.3% in HFpEF and 67.4% in non-HF (*p* < 0.001)). Atrial fibrillation (AF) and chronic kidney disease (CKD) were more prevalent in HFpEF and HFrEF compared to HCM-non-HF (AF: 20.2% and 15.7% versus 6.2% in non-HF, respectively, *p* < 0.001; CKD: 31.7% and 31.0% versus 13.9% in non-HF, respectively, *p* < 0.001). In terms of echocardiographic parameters, patients with HFrEF had the largest left ventricular end-systolic diameter (LVESD), left ventricular end-diastolic diameter (LVEDD) and left atrial (LA) diameter, along with the lowest LVEF (*p* < 0.001). The prevalence of obstructive HCM was significantly lower in patients with HFrEF compared to those with HFpEF or non-HF (*p* < 0.001). The usage of beta-blockers and renin-angiotensin-aldosterone system (RAAS) inhibitors in the HFpEF group was lower compared to the HFrEF group.

**Table 1:** Baseline characteristics and echocardiographic evaluation of the entire cohort and stratified by HF phenotypes

Variables	Total *N* = 3,620	HCM-non-HF *N* = 1,553	HCM-HFpEF *N* = 1,666	HCM-HFrEF *N* = 401	*P*-value
Demographic data					
Age, years	61.4 ± 14.0	59.7 ± 13.4	63.3 ± 14.1	60.5 ± 14.9	< 0.001
Male, n (%)	2439 (67.4%)	1047 (67.4%)	1072 (64.3%)	320 (79.8%)	< 0.001
BMI, kg/m^2^	25 ± 3.8	25.4 ± 3.9	24.7 ± 3.7	24.9 ± 3.7	< 0.001
Comorbidities, *n* (%)					
CHD	648 (17.9%)	281 (18.1%)	259 (15.5%)	108 (26.9%)	< 0.001
PCI	519 (14.3%)	227 (14.6%)	201 (12.1%)	91 (22.7%)	< 0.001
Hypertension	2402 (66.4%)	1056 (68.0%)	1078 (64.7%)	268 (66.8%)	0.139
Diabetes mellitus	903 (24.9%)	413 (26.6%)	391 (23.5%)	99 (24.7%)	0.122
Dyslipidaemia	741 (20.5%)	389 (25%)	274 (16.4%)	78 (19.5%)	< 0.001
Atrial fibrillation	496 (13.7%)	96 (6.2%)	337 (20.2%)	63 (15.7%)	< 0.001
Ischaemic stroke	431 (11.9%)	176 (11.3%)	216 (13.0%)	39 (9.7%)	0.130
Chronic kidney disease	859 (23.7%)	216 (13.9%)	516 (31.0%)	127 (31.7%)	< 0.001
Clinical parameters					
Troponin I	0.3 (0.0–23.3)	0.1 (0.0–14.1)	0.3 (0.0–33.8)	7.0 (0.1–48.0)	< 0.001
NT-proBNP	1115 (365–3111)	218 (94–438)	2147 (1070–4856)	1691(704–4861)	< 0.001
Medicine treatment, *n* (%)					
Diuretic	1417 (39.1%)	301 (19.4%)	838 (50.3%)	278 (69.3%)	< 0.001
Beta-blocker	2174 (60.1%)	916 (59.0%)	1005 (60.3%)	253 (63.1%)	0.311
ACEI/ARB/ARNI	2056 (56.8%)	917 (59.0%)	889 (53.4%)	250 (62.3%)	< 0.001
ACEI	630 (17.4%)	244 (15.7%)	307 (18.4%)	79 (19.7%)	0.056
ARB	1731 (47.8%)	808 (52%)	751 (45.1%)	172 (42.9%)	< 0.001
ARNI	153 (4.2%)	54 (3.5%)	35 (2.1%)	64 (16%)	< 0.001
Calcium-channel blocker	2028 (56%)	909 (58.5%)	919 (55.2%)	200 (49.9%)	0.005
Echocardiographic evaluation					
LV-MWT, mm	17.1 ± 6.8	16.6 ± 7.2	17.5 ± 6.3	17.1 ± 7.3	0.003
LV-MWT ≥ 20mm	588 (16.2%)	210 (13.5%)	338 (20.3%)	40 (10.0%)	< 0.001
LV posterior wall thickness	12.3 ± 2.4	11.9 ± 2.1	12.4 ± 2.4	13 ± 3.0	< 0.001
LVOT obstruction	486 (13.4%)	204 (13.1%)	269 (16.1%)	13 (3.2%)	< 0.001
LVOT gradients at rest, mmHg	32.0 (21.0–59.0)	30.0 (20.0–52.0)	34.0 (22.0–69.0)	26.0 (15.5–59.0)	0.005
E/e’ ratio	13.8 ± 5.6	12.6 ± 4.7	15.1 ± 6.0	13.9 ± 6.2	< 0.001
E/A ratio	0.9 ± 0.6	0.9 ± 0.4	1.0 ± 0.6	1.1 ± 0.7	< 0.001
PASP, mmHg	24.5 ± 3.8	23.9 ± 3.7	24.8 ± 3.8	25.6 ± 4.3	< 0.001
LVEF, %	61.8 ± 9.8	64.9 ± 6.8	63.9 ± 6.6	41.3 ± 6.1	< 0.001
LAD, mm	45.2 ± 6.5	43.4 ± 5.7	46.3 ± 6.6	47.4 ± 7	< 0.001
LVEDD, mm	48.8 ± 7.3	47.8 ± 6.5	48.1 ± 6.8	55.1 ± 9.1	< 0.001
LVESD, mm	32.1 ± 7.0	30.7 ± 5.2	31.2 ± 5.6	41.5 ± 10.2	< 0.001
CO, L	5.2 ± 1.8	5.1 ± 1.6	5.3 ± 1.9	4.9 ± 2.2	0.004
Moderate-severe MR	165 (4.6%)	69 (4.4%)	75 (4.5%)	21 (5.2%)	0.785

Abbreviations: *ACEI* angiotensin converting enzyme inhibitor, *ARBs* angiotensin receptor blocker, *ARNI* angiotensin receptor neprilysin inhibitor, *BMI* body mass index, *CHD* coronary heart disease, *CO* cardiac output, *ES-HF* end-stage heart failure, *HF* heart failure, *HFpEF* heart failure with preserved ejection fraction, *LAD* left atrial diameter, *LV* left ventricular, *LVESD* left ventricular end systolic diameter, *LVEDD* left ventricular end-diastolic dimension, *LVEF* left ventricular ejection fraction, *MWT* maximum wall thickness, *PASP* pulmonary artery systolic pressure, *PCI* percutaneous coronary intervention, *MR* mitral regurgitation

### Association between HFpEF and the development of ES-HF

**Fig. 2:**
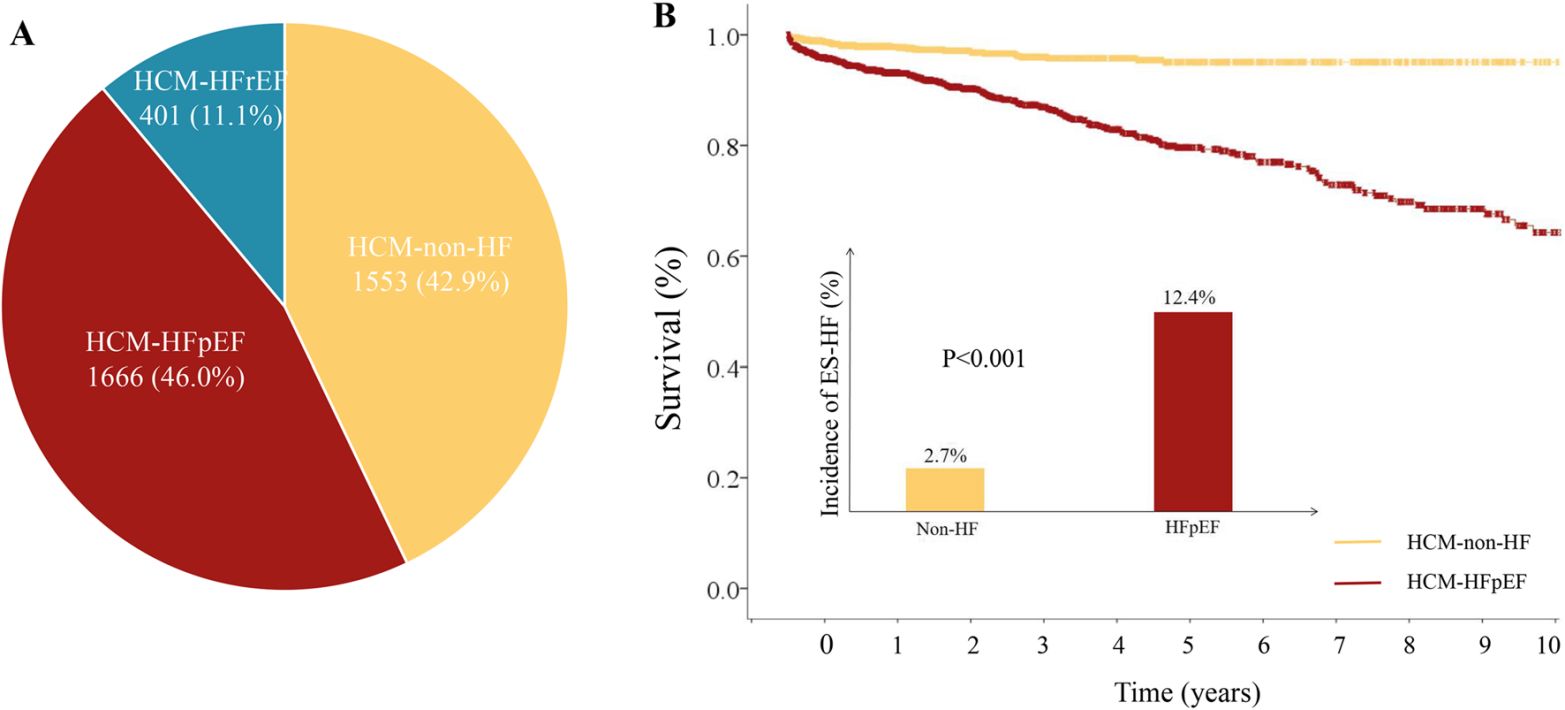
Prevalence of different HF phenotypes in patients with HCM (**A**); Incidence rate of developing ES-HF and Kaplan-Meier curves in HCM patients with normal LVEF stratified by non-HF and HFpEF (**B**). *Abbreviations:* ES-HF: end-stage heart failure; HF: heart failure; HCM: hypertrophic cardiomyopathy; HFpEF, heart failure with preserved ejection fraction; HFrEF: heart failure with reduced ejection fraction; LVEF: left ventricular ejection fraction

In patients with non-HF or HFpEF, 249 (7.7%) patients experienced a decline in LVEF and developed ES-HF (Supplementary Table 1). Kaplan-Meier analysis demonstrated a significant association between HFpEF and ES-HF development compared with non-HF (*P* < 0.001), as shown in Fig. 2. Cox proportional hazards regression analysis showed a strong association between HFpEF and developing ES-HF (HR 4.35, 3.12–6.07, *P* < 0.001) (Table 2). On multivariable analysis adjusted for sex, body mass index, coronary heart disease, prior revascularization, CKD, obstructive HCM, LVEF, and LV dimensions, HFpEF was a significant predictor for the development of ES-HF (HR 3.84, 2.54–5.80, *P* < 0.001). Other significant predictors included prior revascularization (HR 1.82, 1.24–2.69, *P* = 0.002), CKD (HR 2.02, 1.48–2.76, *P* < 0.001), LVESD (HR 1.11, 1.08–1.14, *P* < 0.001).

**Table 2:** Cox regression analyses for predictors of developing ES-HF in HCM patients with normal LVEF ( HCM-non-HF and HCM-HFpEF)

	Univariable analysis		Multivariable analysis	
	HR (95% CI)	*P*-value	HR (95% CI)	*P*-value
Male	1.62 (1.21–2.16)	0.001		
BMI	0.97 (0.93–1.01)	0.138		
HCM-HFpEF vs.HCM-non-HF	4.35 (3.12–6.07)	< 0.001	3.84 (2.54–5.80)	< 0.001
CHD	1.71 (1.27–2.29)	< 0.001		
PCI	1.83 (1.33–2.52)	< 0.001	1.82 (1.24–2.69)	0.002
Chronic kidney disease	2.52 (1.96–3.26)	< 0.001	2.02 (1.48–2.76)	< 0.001
Diuretic	2.81 (2.19–3.61)	< 0.001		
LV posterior wall thickness	1.10 (1.05–1.15)	< 0.001		
LVEF	0.91 (0.89–0.93)	< 0.001		
LAD	1.06 (1.04–1.08)	< 0.001		
LVEDD	1.09 (1.07–1.11)	< 0.001		
LVESD	1.13 (1.10–1.15)	< 0.001	1.11 (1.08–1.14)	< 0.001
CO	1.20 (1.14–1.27)	< 0.001		

Abbreviations: *CHD* coronary heart disease, *CI* confidence interval, *CO* cardiac output, *ES-HF* end-stage heart failure, *HF* heart failure, *HFpEF* heart failure with preserved ejection fraction, *HR* hazard ratio, *LAD* left atrial diameter, *LV* left ventricular, *LVESD* left ventricular end systolic diameter, *LVEDD* left ventricular end-diastolic dimension, *LVEF* left ventricular ejection fraction, *PASP* pulmonary artery systolic pressure, *PCI* percutaneous coronary intervention

## Cumulative incidence of MACEs stratified by HF status

**Fig. 3:**
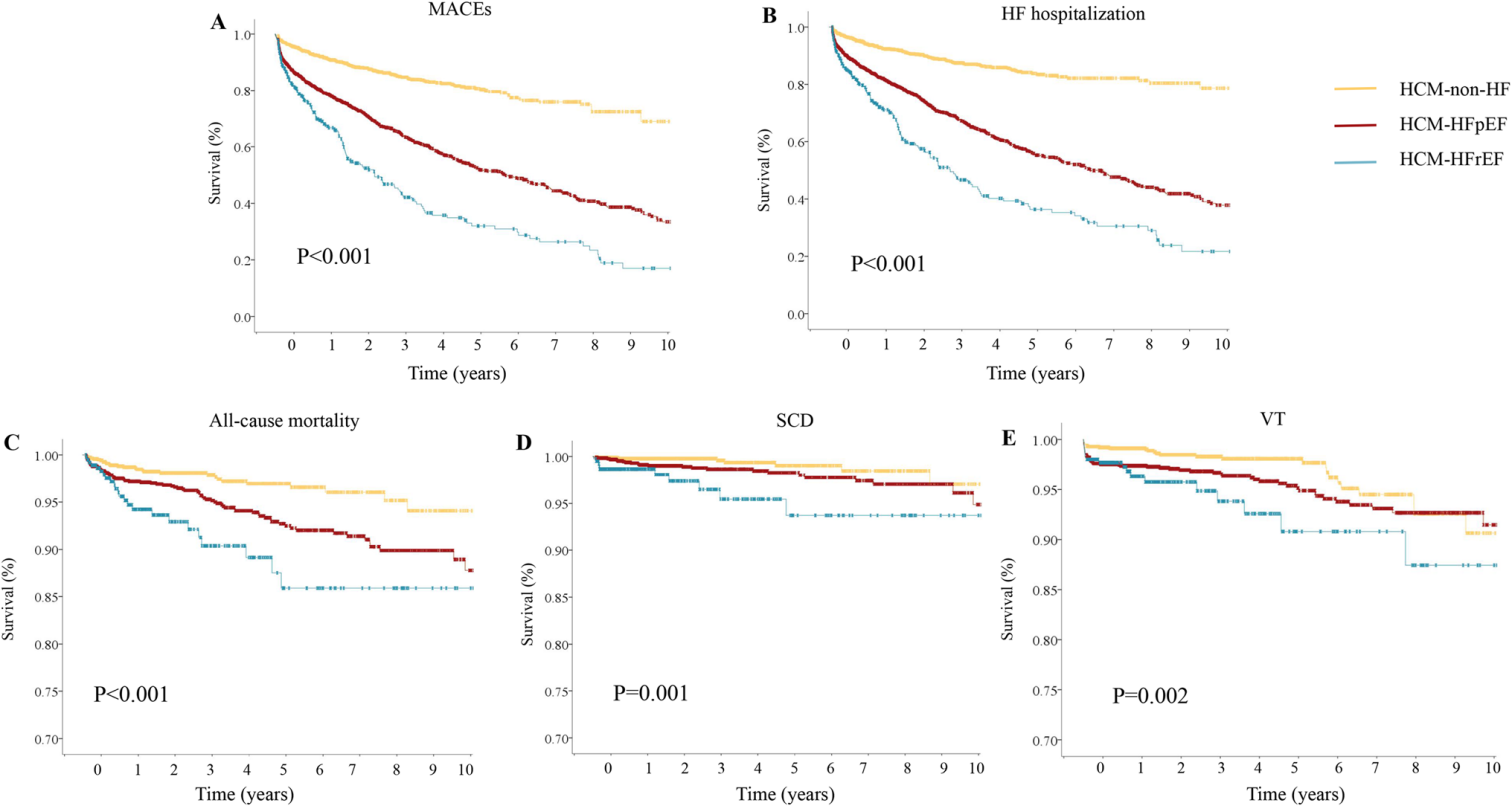
Kaplan-Meier event-free survival curves in patients with HCM stratified by HF phenotypes: (**A**) MACEs; (**B**) HF hospitalization; (**C**) All-cause death; (**D**) SCD and (**E**) VT. *Abbreviations:* ES-HF: end-stage heart failure; HF: heart failure; HCM: hypertrophic cardiomyopathy; HFpEF, heart failure with preserved ejection fraction; HFrEF: heart failure with reduced ejection fraction; MACEs: major adverse clinical events; SCD: sudden cardiac death; VT: ventricular tachycardia

During the 4.0 years (IQR 1.4–9.4 years) follow-up period, 973 MACEs were reported (Table 3). Of these, 138 were all-cause deaths, 848 were HF hospitalization, 42 were SCD and 116 were VT. Comparison between patients that did and did not develop MACEs is presented in Supplementary Table 2. Compared to HCM-non-HF patients, patients with HCM-HFpEF had a higher incidence of MACEs (36.6% vs. 12.2%), all-cause deaths (5.0% vs 2.0%), HF hospitalization (32.8% vs. 9.3%), SCD (1.4% vs. 0.5%) and VT (4.1% vs 1.9%). Kaplan-Meier survival analysis illustrated a significant difference in the cumulative event-free survival rate for MACEs (*P* < 0.001), all-cause deaths (*P* < 0.001), HF hospitalization (*P* < 0.001), SCD (*P* = 0.001) and VT (*P* = 0.002), between patients with HCM-non-HF, HCM-HFpEF and HCM-HFrEF (Fig. 3). On Cox proportional hazard analysis, HCM-HFpEF (HR 2.84, 2.41–3.35, *P* < 0.001) and HCM-HFrEF (HR 4.74, 3.86–5.83, *P* < 0.001) were associated with the risk of MACEs (Table 4). On multivariable analysis adjusted for age, co-morbidities, medications, obstructive HCM, LV wall thickness, LVEF and LV dimensions, HCM-HFpEF (HR 2.13, 1.75–2.59, *P* < 0.001) and HCM-HFrEF (HR 2.67, 2.00–3.58, *P* < 0.001) remained significantly associated with the risk of MACEs. Other significant predictors of MACEs included age (HR 1.02, 1.01–1.03, *P* < 0.001), CKD (HR 1.59, 1.35–1.87, *P* < 0.001), diuretic use (HR 1.76, 1.49–2.08, *P* < 0.001), LV wall thickness (HR 1.06, 1.02–1.09, *P* = 0.002) and pulmonary hypertension (HR 1.03, 1.01–1.05, *P* = 0.002). Beta-blocker use was protective (HR 0.78, 0.67–0.91, *`* = 0.002).

**Table 3:** Clinical outcomes in entire cohort and stratified by HF phenotypes

	Total *N* = 3,620	HCM-non-HF *N* = 1,553	HCM-HFpEF *N* = 1,666	HCM-HFrEF *N* = 401	*P*-value
Follow-up period, years	4.0 (1.4–9.4)	4.1 (1.6–9.4)	4.5 (1.4–10.5)	2.5 (0.9–5.0)	< 0.001
MACEs	973 (26.9%)	189 (12.2%)	610 (36.6%)	174 (43.4%)	< 0.001
All-cause deaths	138 (3.8%)	31 (2.0%)	83 (5.0%)	24 (6.0%)	< 0.001
HF hospitalization	848 (23.4%)	145 (9.3%)	547 (32.8%)	156 (38.9%)	< 0.001
SCD	42 (1.2%)	8 (0.5%)	24 (1.4%)	10 (2.5%)	0.002
VT	116 (3.2%)	30 (1.9%)	68 (4.1%)	18 (4.5%)	0.001

Abbreviations: *HF* heart failure, *MACEs* major adverse cardiac events, *SCD* sudden cardiac death, *VT* ventricular tachyarrhythmia

**Table 4:** Cox regression analyses for predictors of MACEs in whole patients

	Univariable analysis		Multivariable analysis	
	HR (95% CI)	*P*-value	HR (95% CI)	*P*-value
Age	1.02 (1.02–1.03)	< 0.001	1.02 (1.01–1.03)	< 0.001
HCM-HFpEF vs. HCM-non-HF	2.84 (2.41–3.35)	< 0.001	2.13 (1.75–2.59)	< 0.001
HCM-HFrEF vs. HCM-non-HF	4.74 (3.86–5.83)	< 0.001	2.67 (2.00–3.58)	< 0.001
PCI	0.94 (0.77–1.15)	0.560		
Dyslipidaemia	0.91 (0.77–1.08)	0.283		
Atrial fibrillation	1.56 (1.32–1.84)	< 0.001		
Chronic kidney disease	2.07 (1.81–2.36)	< 0.001	1.59 (1.35–1.87)	< 0.001
Diuretic	2.66 (2.34–3.02)	< 0.001	1.76 (1.49–2.08)	< 0.001
Beta-blockers	0.87 (0.77–0.99)	0.030	0.78 (0.67–0.91)	0.002
LV posterior wall thickness	1.08 (1.06–1.11)	< 0.001	1.06 (1.02–1.09)	0.002
PASP	1.05 (1.03–1.07)	< 0.001	1.03 (1.01–1.05)	0.002
LVEF	0.97 (0.96–0.97)	< 0.001		
LAD	1.04 (1.03–1.05)	< 0.001		
LVEDD	1.02 (1.01–1.03)	< 0.001		
LVESD	1.03 (1.02–1.04)	< 0.001		
CO	1.04 (1.01–1.08)	0.022		

Abbreviations: *CI* confidence interval, *CO* cardiac output, *ES-HF* end-stage heart failure, *HF* heart failure, *HFpEF* heart failure with preserved ejection fraction, *HR* hazard ratio, *LAD* left atrial diameter, *LV* left ventricular, *LVESD* left ventricular end systolic diameter, *LVEDD* left ventricular end-diastolic dimension, *LVEF* left ventricular ejection fraction, *MACEs* major adverse clinical events, *PASP* pulmonary artery systolic pressure, *PCI* percutaneous coronary intervention

### Sensitivity analysis

**Fig. 4:**
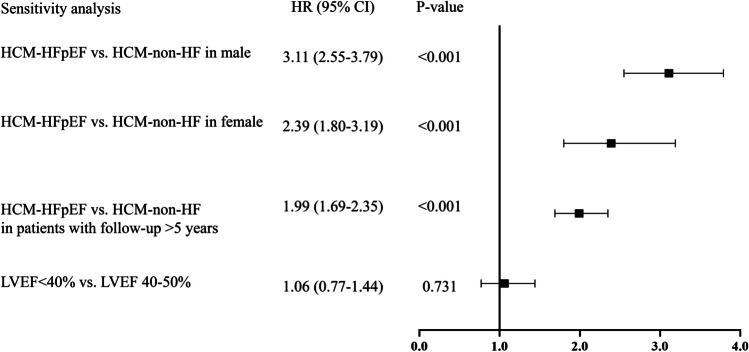
Sensitivity analysis of the association of HCM-HFpEF with MACEs Abbreviations: HCM: hypertrophic cardiomyopathy; HF: heart failure; HFpEF, heart failure with preserved ejection fraction; LVEF: left ventricular ejection fraction

In addition, we conducted several sensitivity analyses to further explore the relationship between HCM-HFpEF and adverse outcomes (Fig. 4). For gender differences in HCM-HFpEF development, HFpEF in both males (HR 3.11, 2.55–3.79, *P* < 0.001) and females (HR 2.39, 1.80–3.19, *P* < 0.001) remained significantly associated with the risk of MACEs. We also performed the sensitivity analysis for patients with follow-up > 5 years. The results indicated that HFpEF remained significantly associated with the risk of MACEs (HR 1.99, 1.69–2.35, *P* < 0.001). When comparing patients with EF < 40% to those with EF 40–50%, there was no statistical difference in reaching the primary endpoint (HR 1.06, 95% CI 0.77–1.44, *P* = 0.731).

## Discussion

In this large contemporary cohort of HCM patients in China, we compared the outcomes of different HF phenotypes (non-HF, HFpEF and HFrEF), analyzed the risk factors for downstream development of ES-HF, and made the following observations:

1. At the time of HCM diagnosis, the prevalence of HFpEF was 46.0% while 11.1% had HFrEF.
2. Patients diagnosed with HCM-HFpEF had a higher risk of developing ES-HF and a poorer prognosis when compared to those with HCM-non-HF.

### Prevalence and clinical characteristics of HFpEF among patients with HCM

Few studies have reported on the prevalence and clinical characteristics of HFpEF in HCM [24]. Liu et al. enrolled a total of 1178 patients with HCM, excluding those with ES-HF, and 513 (43.5%) were identified with HFpEF [24]. Our study similarly found a 46% prevalence of HFpEF. Additionally, we found that patients with HFpEF had distinct clinical attributes compared to non-HF patients. Patients with HFpEF had a higher prevalence of AF, CKD, and larger LA size compared to the non-HF group. We also have made significant observations regarding the predictors for systolic dysfunction in our extensive cohort of HCM patients with preserved EF. Our research suggests that patients who have both PCI and CKD may experience a transitional phase toward ES-HF. CKD is known to increase the risk of worsening heart failure and negatively impact the overall prognosis. Similarly, PCI patients may develop coronary artery stenosis and heart dysfunction, which makes them more prone to developing end-stage heart failure. Therefore, it is crucial to closely monitor these patients for the development of systolic dysfunction. Expectedly, ES-HF had more adverse LV remodeling, with higher use of beta-blockers and RAAS inhibitors.

### HFpEF has an increased risk of developing ES-HF compared to those without HF

ES-HF is linked to poor prognosis, related to myocardial fibrosis, SCD, and refractory HF [25, 26]. A subgroup of patients with HCM, approximately 2%-5% with an incidence rate of 0.5–1.0 patients per 100 patient-years, experience disease progression to ES-HF. We noted that 2.7% of patients with non-HF and 12.4% of patients with HFpEF experienced an LVEF decline and developed ES-HF during the 4.0 years follow-up period. On multivariable analysis, HFpEF was significantly associated with the future development of ES-HF. These observations suggest that a diagnosis of HFpEF may be a predictor of disease progression in HCM patients.

### HFpEF has a poorer prognosis compared to those without HF

There is currently little evidence regarding the clinical and prognostic implications of HFpEF in HCM [20, 24]. Our study demonstrated that patients with HCM-HFpEF had a 2.13-fold increased risk of MACEs compared to those without HF, while patients with HCM-HFrEF had a similar (2.67-fold) increased risk of MACEs compared to those without HF. The identification of patients with HFpEF may aid in the risk stratification of patients with HCM.

### Study limitations

Despite our large HCM cohort, the current study has some important limitations.

First, the single-center observational nature of the analysis has limitations characteristic of this study design. A fixed follow-up interval, such as every 6 or 12 months, can provide a more effective and objective way to evaluate the impact of risk factors on clinical outcomes. This study, on the other hand, is an observational study where patients were not followed up at specific intervals but were instead guided by their cardiologists. As a retrospective study, it may not be possible to avoid certain diagnoses that could have been overlooked. Asymptomatic heart failure may go unrecognized and undiagnosed, leading to potential bias. Second, objective stress tests may raise concerns about diagnostic consistency and the reliability of the study's conclusions, but these were not routine measurements in this retrospective study. Third, genetic testing and late gadolinium enhancement (LGE) on cardiac MRI have the ability to expand the spectrum of the HCM disease and identify HCM phenocopies with varying natural histories. Unfortunately, these tests are not available to most patients, which can result in HCM being overlooked during diagnosis. Fourth, this study was an observational study, and patients who underwent Holter or ECG tests were mainly guided by cardiologists, leading to a lack of information on non-sustained ventricular tachycardia (NSVT) or Holter monitoring at fixed intervals.

Last, this cohort included a Chinese population and as such results may not be generalizable to other ethnic groups.

## Conclusions

HFpEF is frequently observed in patients with HCM. Patients with HFpEF are at a higher risk of experiencing a decline in LVEF, emphasizing the need for close echocardiography follow-up. Additionally, HFpEF confers a worse prognosis compared to those without HF, and may aid in risk stratification.

### Supplementary Information

Below is the link to the electronic supplementary material.

Supplementary Table 1 Baseline characteristics and echocardiographic evaluation in HCM patients with normal LVEF stratified by developing ES-HF or not (PDF 222 KB)

Supplementary Table 2 Baseline characteristics and echocardiographic evaluation in HCM patients stratified by developing MACEs or not. (PDF 268 KB)
