# Supplementary material for: Clinical characteristics and prognosis of patients with hypertrophic cardiomyopathy and heart failure with preserved ejection fraction
Source: Clin Res Cardiol. 2024 Jan 10;113(5):761–9. doi: 10.1007/s00392-023-02371-5 (PMC11026190; doi:10.1007/s00392-023-02371-5)
Supplement: Supplementary file 1 — Supplementary Table 1 Baseline characteristics and echocardiographic evaluation in HCM patients with normal LVEF stratified by developing ES-HF or not (PDF 222 KB) [file 392_2023_2371_MOESM1_ESM.pdf]

Supplementary Table 1. Baseline characteristics and echocardiographic evaluation in HCM patients with normal LVEF stratified by developing ES-HF or not

Variables	Total N=3,219	Non-ES-HF N=2,970	ES-HF N=249	P-value
**Demographic data**				
Age, years	61.6±13.9	61.6±13.8	60.6±14.8	0.261
Male, n (%)	2119 (65.8%)	1931 (65.0%)	188 (75.5%)	0.001
BMI, kg/m^2	25.0±3.8	25.1±3.8	24.6±3.7	0.047
**HF status**				<0.001
Non-HF	1553 (48.2%)	1511 (50.9%)	42 (16.9%)	
HFpEF	1666 (51.8%)	1459 (49.1%)	207 (83.1%)	
**Comorbidities, n (%)**				
CHD	540 (16.8%)	483 (16.3%)	57 (22.9%)	0.007
PCI	428 (13.3%)	381 (12.8%)	47 (18.9%)	0.007
Hypertension	2134 (66.3%)	1983 (66.8%)	151 (60.6%)	0.050
Diabetes mellitus	804 (25.0%)	740 (24.9%)	64 (25.7%)	0.783
Dyslipidaemia	663 (20.6%)	619 (20.8%)	44 (17.7%)	0.235
Atrial fibrillation	433 (13.5%)	402 (13.5%)	31 (12.4%)	0.630
Ischaemic stroke	392 (12.2%)	354 (11.9%)	38 (15.3%)	0.121
Chronic kidney disease	732 (22.7%)	635 (21.4%)	97 (39.0%)	<0.001
**Clinical parameters**				
Troponin I	0.2 (0.0-20.7)	0.2 (0.0-19.6)	0.5 (0.1-56.1)	<0.001
NT-proBNP	1026 (330-2961)	923 (292-2549)	2990 (961-8075)	<0.001
**Medicine treatment, n (%)**				
Diuretic	1139 (35.4%)	1010 (34.0%)	129 (51.8%)	<0.001
Beta-blocker	1921 (59.7%)	1786 (60.1%)	135 (54.2%)	0.067
ACEI/ARB/ARNI	1806 (56.1%)	1677 (56.5%)	129 (51.8%)	0.155
ACEI	551 (17.1%)	494 (16.6%)	57 (22.9%)	0.015
ARB	1559 (48.4%)	1450 (48.8%)	109 (43.8%)	0.143
ARNI	89 (2.7%)	84 (2.8%)	5 (2%)	0.577
Calcium-channel blocker	1828 (56.8%)	1696 (57.1%)	132 (53.0%)	0.211
**Echocardiographic evaluation**				
LV-MWT, mm	17.1±6.8	17.1±6.7	16.8±7.5	0.465
LV-MWT ≥20mm	548 (17.0%)	513 (17.3%)	35 (14.1%)	0.195
LV posterior wall thickness	12.2±2.3	12.1±2.2	12.5±2.6	0.008
LVOT obstruction	473 (14.7%)	456 (15.4%)	17 (6.8%)	<0.001
LVOT gradients at rest, mmHg	32.0 (21.0-59.0)	32.0 (21.0-59.0)	32.0 (19.0-61.5)	0.774
E/e	13.8 ± 5.5	13.7 ± 5.5	14.7 ± 5.7	0.139
E/A	0.9 ± 0.5	0.9 ± 0.5	1.2 ± 0.9	0.006
PASP, mmHg	24.4±3.8	24.3±3.7	25.2±4.4	0.001
LVEF, %	64.4±6.7	64.7±6.6	60.5±6.4	<0.001
LAD, mm	44.9±6.4	44.7±6.3	46.8±7.2	<0.001
LVEDD, mm	48.0±6.7	47.7±6.5	51.9±7.7	<0.001
LVESD, mm	31.0±5.4	30.7±5.2	34.8±6.6	<0.001

CO, L	5.2±1.8	5.1±1.7	5.9±2.3	<0.001
Moderate-severe MR	144 (4.5%)	131 (4.4%)	13 (5.2%)	0.553

Abbreviations: ACEI: angiotensin converting enzyme inhibitor; ARBs: angiotensin receptor blocker; ARNI: angiotensin receptor neprilysin inhibitor; BMI: body mass index; CHD: coronary heart disease; CO: cardiac output; ES-HF: end-stage heart failure; HF: heart failure; HFpEF, heart failure with preserved ejection fraction; LAD: left atrial diameter; LV: left ventricular; LVESD: left ventricular end systolic diameter; LVEDD: left ventricular end-diastolic dimension; LVEF: left ventricular ejection fraction; MWT: maximum wall thickness; PASP: pulmonary artery systolic pressure; PCI: percutaneous coronary intervention; MR: mitral regurgitation.
