# Supplementary material for: Clinical characteristics and prognosis of patients with hypertrophic cardiomyopathy and heart failure with preserved ejection fraction
Source: Clin Res Cardiol. 2024 Jan 10;113(5):761–9. doi: 10.1007/s00392-023-02371-5 (PMC11026190; doi:10.1007/s00392-023-02371-5)
Supplement: Supplementary file 2 — Supplementary Table 2 Baseline characteristics and echocardiographic evaluation in HCM patients stratified by developing MACEs or not. (PDF 268 KB) [file 392_2023_2371_MOESM2_ESM.pdf]

Supplementary Table 2. Baseline characteristics and echocardiographic evaluation in HCM patients stratified by developing MACEs or not

Variables	Total N=3,620	Non-MACEs N=2,647	MACEs N=973	P-value
**Demographic data**				
Age, years	61.4±14	60.6±13.9	63.8±13.9	<0.001
Male, n (%)	2439 (67.4%)	1769 (66.8%)	670 (68.9%)	0.248
BMI, kg/m^2	25.0±3.8	25.1±3.8	24.8±3.7	0.060
**HF status**				<0.001
Non-HF	1553 (42.9%)	1364 (51.5%)	189 (19.4%)	
HFpEF	1666 (46.0%)	1056 (39.9%)	610 (62.7%)	
ES-HCM	401 (11.1%)	227 (8.6%)	174 (17.9%)	
**Comorbidities, n (%)**				
CHD	648 (17.9%)	492 (18.6%)	156 (16.0%)	0.076
PCI	519 (14.3%)	407 (15.4%)	112 (11.5%)	0.003
Hypertension	2402 (66.4%)	1779 (67.2%)	623 (64%)	0.073
Diabetes mellitus	903 (24.9%)	656 (24.8%)	247 (25.4%)	0.710
Dyslipidaemia	741 (20.5%)	578 (21.8%)	163 (16.8%)	0.001
Atrial fibrillation	496 (13.7%)	333 (12.6%)	163 (16.8%)	0.001
Ischaemic stroke	431 (11.9%)	308 (11.6%)	123 (12.6%)	0.408
Chronic kidney disease	859 (23.7%)	512 (19.3%)	347 (35.7%)	<0.001
**Clinical parameters**				
Troponin I	0.3 (0.0-23.3)	0.3 (0.0-22.0)	0.2 (0.0-28.9)	0.136
NT-proBNP	1115 (365-3111)	759 (240-2224)	2192 (989-5499)	<0.001
**Medicine treatment, n (%)**				
Diuretic	1417 (39.1%)	883 (33.4%)	534 (54.9%)	<0.001
Beta-blocker	2174 (60.1%)	1650 (62.3%)	524 (53.9%)	<0.001
ACEI/ARB/ARNI	2056 (56.8%)	1523 (57.5%)	533 (54.8%)	0.138
ACEI	630 (17.4%)	443 (16.7%)	187 (19.2%)	0.090
ARB	1731 (47.8%)	1293 (48.8%)	438 (45%)	0.045
ARNI	153 (4.2%)	128 (4.8%)	25 (2.6%)	0.004
Calcium-channel blocker	2028 (56.0%)	1486 (56.1%)	542 (55.7%)	0.815
**Echocardiographic evaluation**				
LV-MWT, mm	17.1±6.8	17.1±7.4	16.9±5.2	0.403
LV-MWT ≥20mm	588 (16.2%)	431 (16.3%)	157 (16.1%)	0.915
LV posterior wall thickness	12.3±2.4	12.1±2.3	12.6±2.5	<0.001
LVOT obstruction	486 (13.4%)	382 (14.4%)	104 (10.7%)	0.003
LVOT gradients at rest, mmHg	32.0 (21.0-59.0)	32.0 (20.0-56.0)	34.0 (23.0-67.0)	0.104
E/e	13.8 ± 5.6	13.4 ± 5.2	15.2 ± 6.6	<0.001
E/A	0.9 ± 0.6	0.9 ± 0.5	1.0 ± 0.7	0.018
PASP, mmHg	24.5±3.8	24.2±3.8	25.3±3.9	<0.001
LVEF, %	61.8±9.8	62.6±9.3	59.7±10.9	<0.001
LAD, mm	45.2±6.5	44.7±6.3	46.5±6.8	<0.001
LVEDD, mm	48.8±7.3	48.4±7.1	49.8±7.9	<0.001

LVESD, mm	32.1±7.0	31.6±6.5	33.5±8.0	<0.001
CO, L	5.2±1.8	5.1±1.8	5.3±2.0	0.014
Moderate-severe MR	165 (4.6%)	122 (4.6%)	43 (4.4%)	0.808

Abbreviations: ACEI: angiotensin converting enzyme inhibitor; ARBs: angiotensin receptor blocker; ARNI: angiotensin receptor neprilysin inhibitor; BMI: body mass index; CHD: coronary heart disease; CO: cardiac output; ES-HF: end-stage heart failure; HF: heart failure; HFpEF, heart failure with preserved ejection fraction; LAD: left atrial diameter; LV: left ventricular; LVESD: left ventricular end systolic diameter; LVEDD: left ventricular end-diastolic dimension; LVEF: left ventricular ejection fraction; MWT: maximum wall thickness; PASP: pulmonary artery systolic pressure; PCI: percutaneous coronary intervention; MR: mitral regurgitation.
